# Supplementary material for: Genetic analysis of blood molecular phenotypes reveals common properties in the regulatory networks affecting complex traits
Source: Nat Commun. 2023 Aug 21;14:5062. doi: 10.1038/s41467-023-40569-3 (PMC10442420; doi:10.1038/s41467-023-40569-3)
Supplement: Supplementary file 1 — Supplementary Information [file 41467_2023_40569_MOESM1_ESM.pdf]

Supplementary Figures and Tables for

Genetic analysis of blood molecular phenotypes reveals common properties in the regulatory networks affecting complex traits.

Andrew A. Brown[1], Juan J. Fernandez-Tajes[2], Mun-gwan Hong[3], Caroline A. Brorsson[4,5], Robert W. Koivula[6], David Davtian[1], Théo Dupuis[1], Ambra Sartori[7,8,9], Theodora-Dafni Michalettou[10], Ian M. Forgie[1], Jonathan Adam[11,12], Kristine H. Allin[13], Robert Caiazzo[14], Henna Cederberg[15], Federico De Masi[4], Petra J.M. Elders[16], Giuseppe N. Giordano[17], Mark Haid[18], Torben Hansen[13], Tue H. Hansen[13], Andrew T. Hattersley[19], Alison J. Heggie[20], Cédric Howald[7,8,9], Angus G. Jones[19], Tarja Kokkola[15], Markku Laakso[15], Anubha Mahajan[2], Andrea Mari[21], Timothy J. McDonald[22], Donna McEvoy[23], Miranda Mourby[24], Petra B. Musholt[25], Birgitte Nilsson[4], Francois Pattou[14], Deborah Penet[7,8,9], Violeta Raverdy[14], Martin Ridderstråle[26], Luciana Romano[7,8,9], Femke Rutters[27], Sapna Sharma[12,28], Harriet Teare[29], Leen 't Hart[27,30,31], Konstantinos D. Tsirigos[4], Jagadish Vangipurapu[15], Henrik Vestergaard[13,32], Søren Brunak[4,5], Paul W. Franks[17], Gary Frost[33], Harald Grallert[11,12], Bernd Jablonka[34], Mark I McCarthy[2,35], Imre Pavo[36], Oluf Pedersen[37,38], Hartmut Ruetten[34], Mark Walker[39], The DIRECT Consortium, Jerzy Adamski[40,41,42], Jochen M. Schwenk[3], Ewan R. Pearson[1], Emmanouil T. Dermitzakis[7,8,9,*,+], Ana Viñuela[10,*,+]

[*] Corresponding authors: Ana Viñuela (ana.vinuela@newcastle.ac.uk) and Emmanouil T. Dermitzakis (emmanouil.dermitzakis@unige.ch).

[+] These authors contributed equally.

This PDF file includes:

Tables S1 to S2

Figs. S1 to S10

All supplementary datasets are included in the following link:
https://zenodo.org/record/7521410

Phenotype	N. Samples	Male/Female	N. Phenotypes
**Genotypes**	3,029	2142/887	9,472,419
**Gene expression**	3,029	2142/887	16,205
**Exon expression**	3,029	2142/887	140,198
**Splicing phenotypes**	3,029	2142/887	64,546
**Targeted proteome**	3,027	2141/886	373
**Targeted metabolites**	3,029	2142/887	116
**Untargeted metabolites**	2,998	2121/887	233

Supplementary Table 1 | After quality evaluation of all molecular phenotypes, the full dataset was restricted to individuals with complete RNAseq and genotypes data (N=3,029). Full break down of the number of participants for study with the different molecular phenotypes is included here. All analyses were performed with the maximum possible number of participants, which oscillates between 2,998 and 3,029.

Phenotype class	Phenotypes	At least 1 association	More than 2 associations	Total QTLs
Cis-eQTLs (genes)	16,205	15,305 (94.4%, π_1=0.968)	12,824	59,972
Cis-eQTLs (exons)	140,198 (15,712)	14,759 (93.9%, π_1 = 0.968)	12,477	59,952
Cis-sQTLs (splicing)	64,546	3,609 (5.59%, π_1=0.655)	298	3,978
Cis-pQTLs (targeted Proteins)	373	363 (97.31% π_1=0.98)	315	1,590
Trans-eQTLs (genes)	15,114	1,670 (11.04%, π_1=0.513)	344	2,320
Trans-pQTLs (targeted proteins)	373	139 (37.2%, π_1=0.463)	95	533
metaboQTLs (targeted metabolites)	116	69 (65.09%, π_1=0.813)	33	102
metaboQTLs (untargeted metabolites)	233	103 (44.2%, π_1=0.535)	96	199

Supplementary Table 2 | QTL discovered in DIRECT. Number of significant QTL (FDR<5%) identified for the different molecular phenotypes. From RNAseq data we derived gene expression quantifications, exon expression quantifications and splicing phenotypes. In addition, we used targeted proteins, targeted metabolites and untargeted metabolites. The analyses were done considering nearby SNPs (cis-QTLs) for expression and protein phenotypes with a genomics location, and considering SNPs acting in trans (more than 5Mb from TSS of the phenotypes). Metabolites, with no specific genomic location were assessed for associations with all the SNPs. The columns indicate the type of phenotypes (Phenotype class) and QTLs derived, the number of phenotypes (Phenotypes) tested in each analysis, the number of phenotypes with at least one significant association, the number of phenotypes with more that two significant associations after performing the conditional association analysis, and the final total number of associations (QTLs) per phenotype.

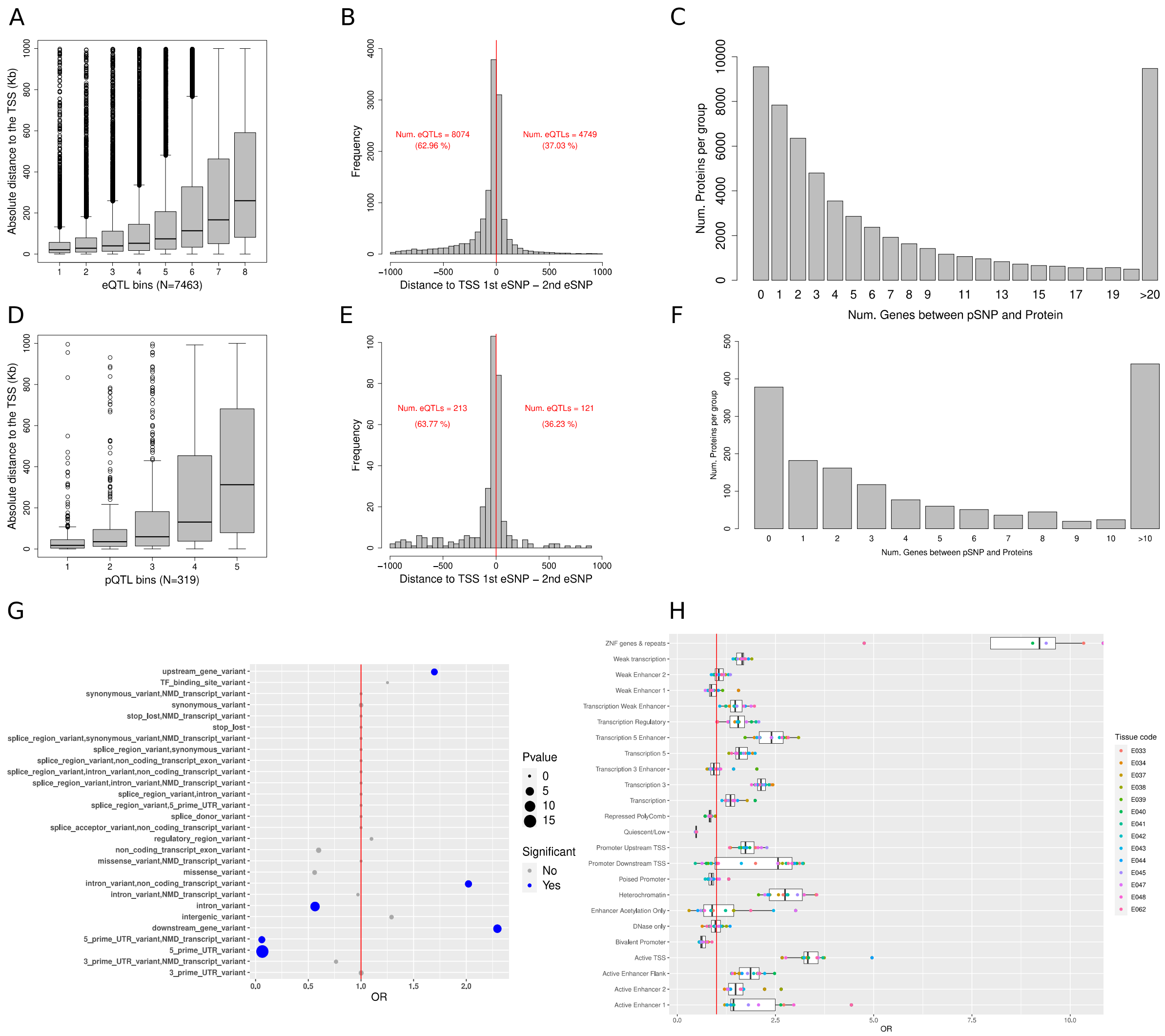

Supplementary Figure 1 | Properties of cis-eQTLs and cis-pQTLs are similar but functional enrichment of eSNP and pSNP suggest differences in activity. A) Primary gene level cis-eQTLs, identify as those cis-eQTLs with the most significant (FDR<0.05) association per gene, were significantly closer from the canonical TSS than secondary signals (Wilcox test = 7.34e-173). The plot shows groups of 7,463 eSNPs with similar Pvalue ordered by their absolute distance between the eSNPs and the TSS (y-axis): group 1 had the largest Pvalues, while group 8 had the smaller, but significant Pvalues. Box plots show the median distance to TSS in boxes defined by the limits of the first and last quartiles as reported by the boxplot() function in R[1]. **B)** The histogram show the distance between stronger (primary) and the second strongest eSNPs for genes with two cis-eQTLs. Positive distance indicates the most significant cis-eQTLs (37.03% of cis-eQTLs) was further way form the TSS of the gene than the second. **C)** Barplot showing the number of other genes which canonical TSS was located between the eSNP and the genes with cis-eQTLs. For 84.66% of the cis-eQTLs, we would identify at least one gene (as defined by Gencode v19) located between the variant and the gene of the cis-eQTL. The bar on the right is grouping all the cis-eQTLs (n=9,476) for which we would find 20 or more genes in between the eGene and the eSNP, with a total range from 0 to 197 genes. **D)** Proteins with at least two cis-pQTLs presented secondary associations closer to the TSS than the primary associations.

The proportion were of 38.46% cis-pQTLs (120) were the secondary pQTLs was closer to the TSS than the primary cis-pQTL. Box plots show the median distance to TSS in boxes defined by the limits of the first and last quartiles as defined by the boxplot() function in R. **E)** Histogram showing the distance between stronger (primary) and the second strongest pSNPs for proteins with two cis-pQTLs. Positive distance indicates the most significant cis-pQTLs (36.23% of cis-pQTLs) was further way from the TSS of the gene than the second. **F)** Barplot showing the number of genes with a canonical TSS located between the pSNP and the genes coding for the protein of the cis-pQTLs. For 78.05% of the cis-pQTLs, we would identify at least one coding gene (as defined by Gencode v19) located between the variant and the gene of the cis-pQTL. **G)** Functional enrichment analysis of cis-eSNP (n=57,200) vs. cis-pSNPs (n=1,580) using VEP. Positive OR indicates enrichment in cis-eSNPs. Pvalues were calculated using a two-sided fisher test. **H)** Functional enrichment analysis of all SNPs acting as cis-eQTLs (n=57,200) compared to SNPs acting as cis-pQTLs (n=1,580) using ChromHMM experiments. Cell types included were: E062 (Primary mononuclear cells from peripheral blood), E034 (Primary T cells from peripheral blood), E045 (Primary T cells effector/memory enriched from peripheral blood), E033 (Primary T cells from cord blood), E044 (Primary T regulatory cells from peripheral blood), E043 (Primary T helper cells from peripheral blood), E039 (Primary T helper naive cells from peripheral blood), E041 (Primary T helper cells PMA-I stimulated), E042 (Primary T helper 17 cells PMA-I stimulated), E040 (Primary T helper memory cells from peripheral blood), E037 (Primary T helper memory cells from peripheral blood), E048 (Primary T CD8+ memory cells from peripheral blood), E038 (Primary T helper naive cells from peripheral blood), and E047 (Primary T CD8+ naive cells from peripheral blood). Box plots show the median distance to TSS in boxes defined by the limits of the first and last quartiles as defined by ggplot[2]. Pvalues were calculated using a two-sided fisher test.

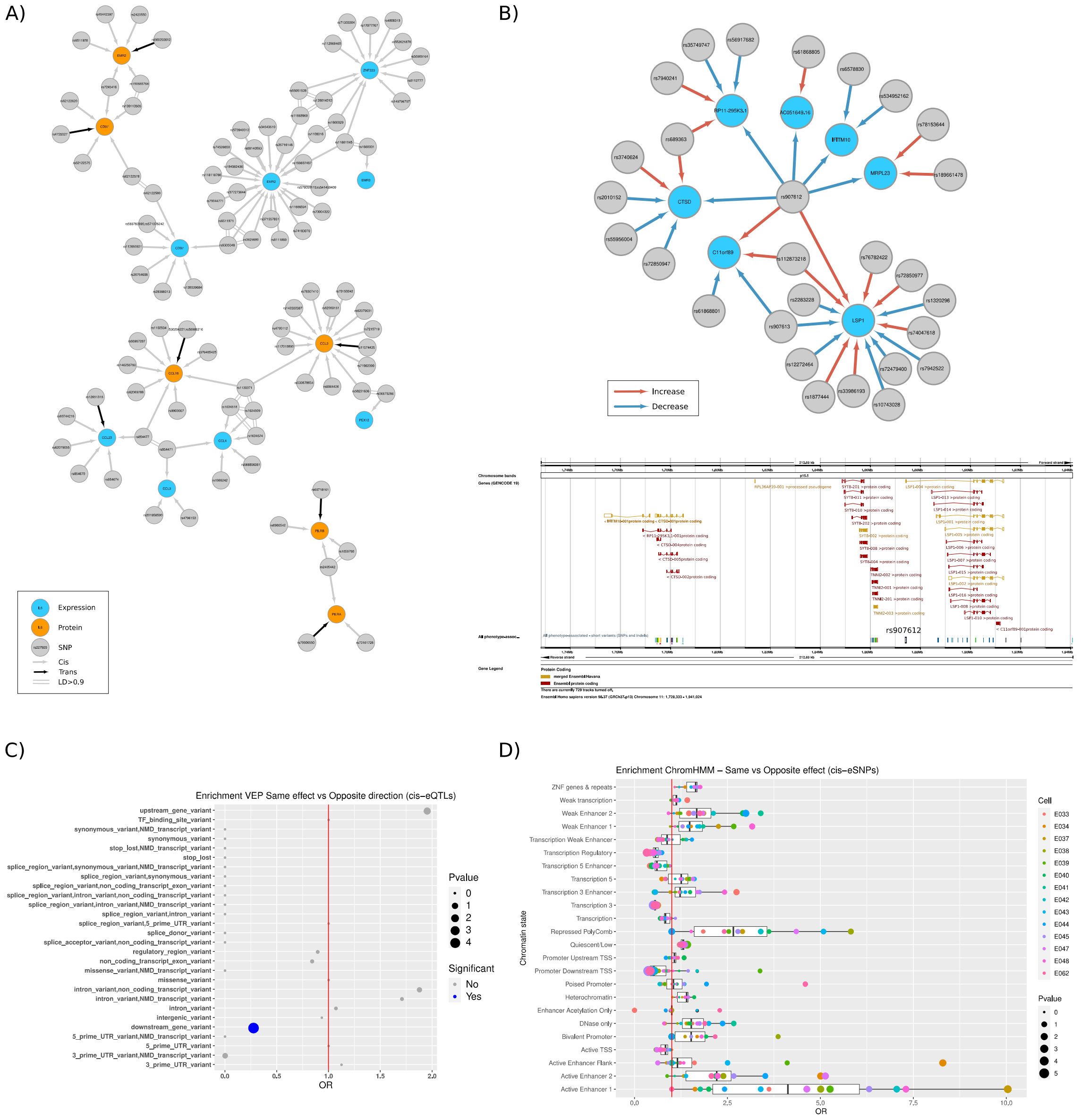

Supplementary Figure 2 | Pleiotropy in cis-QTLs. A) Networks of cis-pSNPs signficantly associated (FDR<0.05, linear regression) to more than one protein. The figure shows the independent networks associated to the pSNPs. Grey arrows indicate significant cis-QTLs for proteins or genes, black arrows identify trans-QTLs. Sample size for the associations was n=3,027. **B)** Example of shared cis-eQTLs (rs907612) involving multiple genes were the same allele had a different direction of effects in chromosome 11. Examples of the effect allele T (rs907612-T) increasing expression (red arrows) of *LSP1* and *C11orf89,* while the same allele degreased the expression (blue arrows) of *IFITM10*, *CTSD*, *MRPL12*, *RP11-295K3.1*, *AC051649.16.* The genomic region, indicated at the bottom, shows the location of rs907612 (black) and other SNPS. Samples size for cis-eQTL was n=3,029. **C)** Functional enrichment analysis of cis-eSNP (n=497) associated to two or more genes with opposite direction of effect and compared to eSNPs (n=1,956) with same direction of effect using VEP. Negative OR indicates enrichment in cis-eSNPs with opposite direction of effect.

Pvalues were calculated using a two-sided fisher test. **D)** Functional enrichment analysis using ChromHMM experiments for eSNPs associated with more than two genes with opposite direction of effect and compared to eSNPs with same direction of effect. Negative OR indicates enrichment in cis-eSNPs with opposite direction of effect. Cell types included were: E062 (Primary mononuclear cells from peripheral blood), E034 (Primary T cells from peripheral blood), E045 (Primary T cells effector/memory enriched from peripheral blood), E033 (Primary T cells from cord blood), E044 (Primary T regulatory cells from peripheral blood), E043 (Primary T helper cells from peripheral blood), E039 (Primary T helper naive cells from peripheral blood), E041 (Primary T helper cells PMA-I stimulated), E042 (Primary T helper 17 cells PMA-I stimulated), E040 (Primary T helper memory cells from peripheral blood), E037 (Primary T helper memory cells from peripheral blood), E048 (Primary T CD8+ memory cells from peripheral blood), E038 (Primary T helper naive cells from peripheral blood), and E047 (Primary T CD8+ naive cells from peripheral blood). Box plots show the median distance to TSS in boxes defined by the limits of the first and last quartiles as defined by ggplot[2]. Pvalues were calculated using a two-sided fisher test.

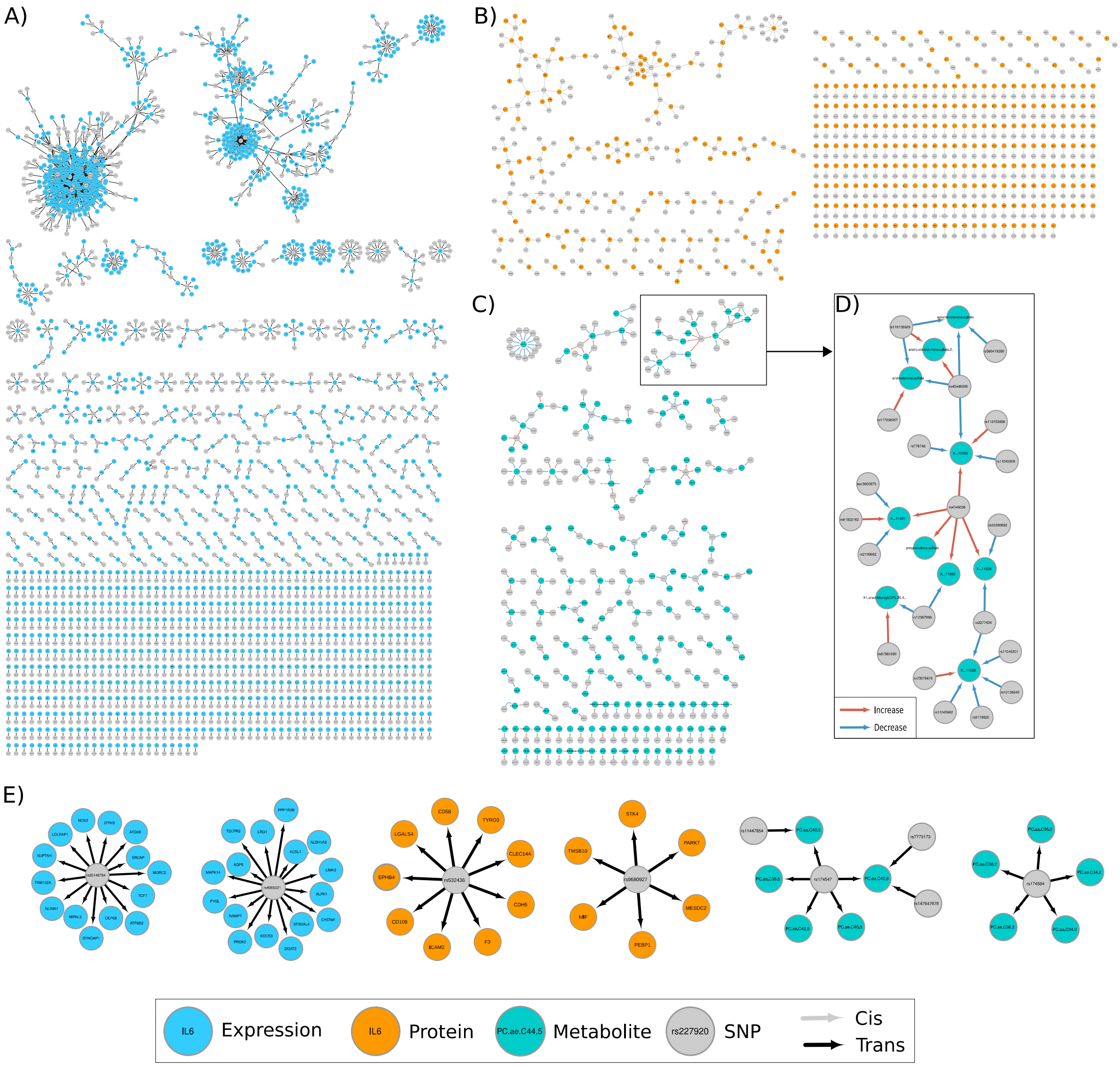

Supplementary Figure 3 | Visualization of significant trans-QTLs. A) Genes are represented as blue nodes while trans-eSNPs (lead SNPs associated to the expression of a gene in trans) are grey nodes. We identified 2,320 independent trans-eQTLs (Supplementary Data 7), involving 1,670 genes and 1,351 SNPs (from the 15,115 genes tested). Of the genes with a trans-eQTL (trans-eGenes), 345 (20.65%) had more than one trans-eSNP independently regulating their expression (mean = 1.39), with a maximum of 13 distal eSNP effects for the expression of *F7*. The SNP with the largest number of associations in trans (top left), was rs1354034, a replicated trans-acting SNP previously associated to platelet counts (Supplementary Figure 10B). **B)** This visualization of trans-pQTL present proteins as orange nodes and trans-pSNPs (lead SNPs associated to the proteins in trans) as grey nodes. Our analysis identified 533 independent trans-pQTLs for 193 proteins. We would observe a similar abundance of allelic heterogeneity than for trans expression associations. The number of proteins being regulated by more than one trans-pSNP was slightly larger than observed for expression. **C)** A genome-wide scan of metabo-

QTLs identified 301 independent associations for 172 targeted and untargeted metabolites (37.47%). Of the associated metabolites 68 have more than one metabo-QTLs (39.5%, mean=1.75), with up to 13 SNPs for 2-piperidinone. Likewise with other molecular phenotypes, we identify variants (n=42) associated to two or more metabolites (mean=1.27). **D)** Highlight of one of the networks derived from metabo-QTLs with a SNP associated with up to 5 metabolites (rs174547). The network shows how the effective allele of a SNPs associated to multiple molecular phenotypes may show opposite directions of effect, stressing the need to understand the genetic regulation of multiple phenotypes from the same datasets. **E)** Examples of trans-SNPs associated to multiple phenotypes. From left to right these include a trans-eQTLs association for rs35148784 with the expression of 14 genes (blue nodes), and associations in trans for rs4065321 with the expression of 16 genes, associations for rs532436 with 9 proteins (orange nodes) and rs9680927 and 6 proteins, and metabo-QTL associations for rs174547 and rs174584 with 4 and 5 metabolites.

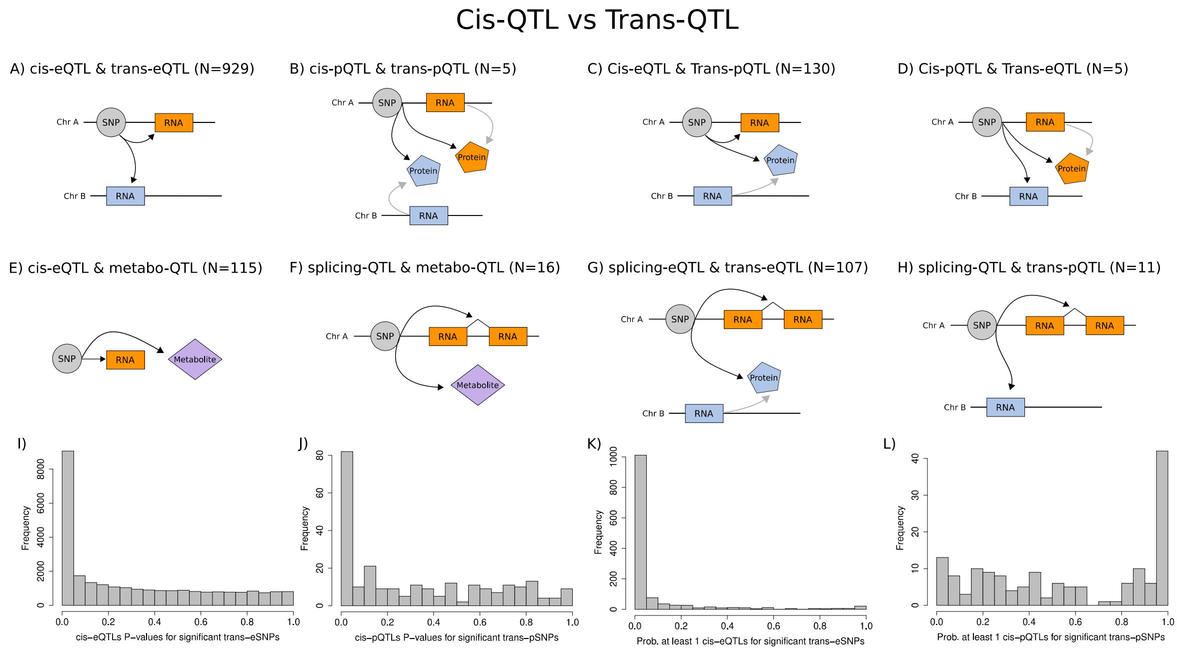

lementary Figure 4 | cis-SNPs acting also as trans-SNPs. From the significant QTL associations, we reported how many SNPs acted both locally (in cis) and distantly (in trans). We considered also pairs of SNPs in high LD (R^2>0.9). Metabolite-QTLs (metabo-QTL) were considered as trans-QTLs. Each of the diagrams show the SNP-phenotype pairs with multiple cis- and trans-QTLs. **A)** The number of trans-eQTLs that shared the same SNPs with cis-eQTLs were 262, including SNPs in high LD we identified 929 cases. **B)** Cis-pQTL that were also trans-pQTLs were 5, although we found no case were the exact same SNP acted both in cis and trans. **C)** SNPs acting on local gene expression and distal protein levels were 130 (26 reported same lead SNP). **D)** We identified 5 trans-eQTLs that were also cis-pQTLs, including rs34097845, a SNP in chromosome 17 associated locally with the MPO protein and acting distally to regulate the expression of both *HSPA5* (chromosome 9) and *HSP90B1* (chromosome 12) (Figure 1K). **E)** Cis-eQTLs that were also acting as metabo-QTL were 115. **F)** splicing-QTLs shared associations with 16 metabo-QTLs; **G)** 107 associations with trans-eQTLs, and **H)** 11 with trans-pQTLs. **I)** Distribution of all cis-eQTL associations p-values from linear regression for expression-SNPs pairs of significant trans-eSNPs. The π_1 enrichment using all Pvalues (n=26,956) was of 41.42% for trans-eSNPs acting as cis-eQTLs. **J)** Distribution using all cis-pQTL Pvalues (n=253) from linear regression for protein-SNPs pairs of significant trans-pSNPs. The π_1 enrichment was in this case of 41.68% for trans-pSNPs acting as cis-pQTLs. **K)** To account for multiple cis-QTL associations, we calculated the probability of trans-eSNPs being a cis-eQTL for at least one gene. The graph shows the product of Pvalues per gene as the probability of one gene to be also a trans-eSNP (n=2,600). The π_1 enrichment identify 77.34% trans-eSNPs also acting as cis-SNP for at least one gene. **L)** Similarly to eQTLs, we plot the product of Pvalues per protein as the probability of one protein to be also a trans-pSNP for at least one protein (n=153). The π_1 enrichment was of 0%, identifying no trans-pSNPs acting also as cis-pQTLs.

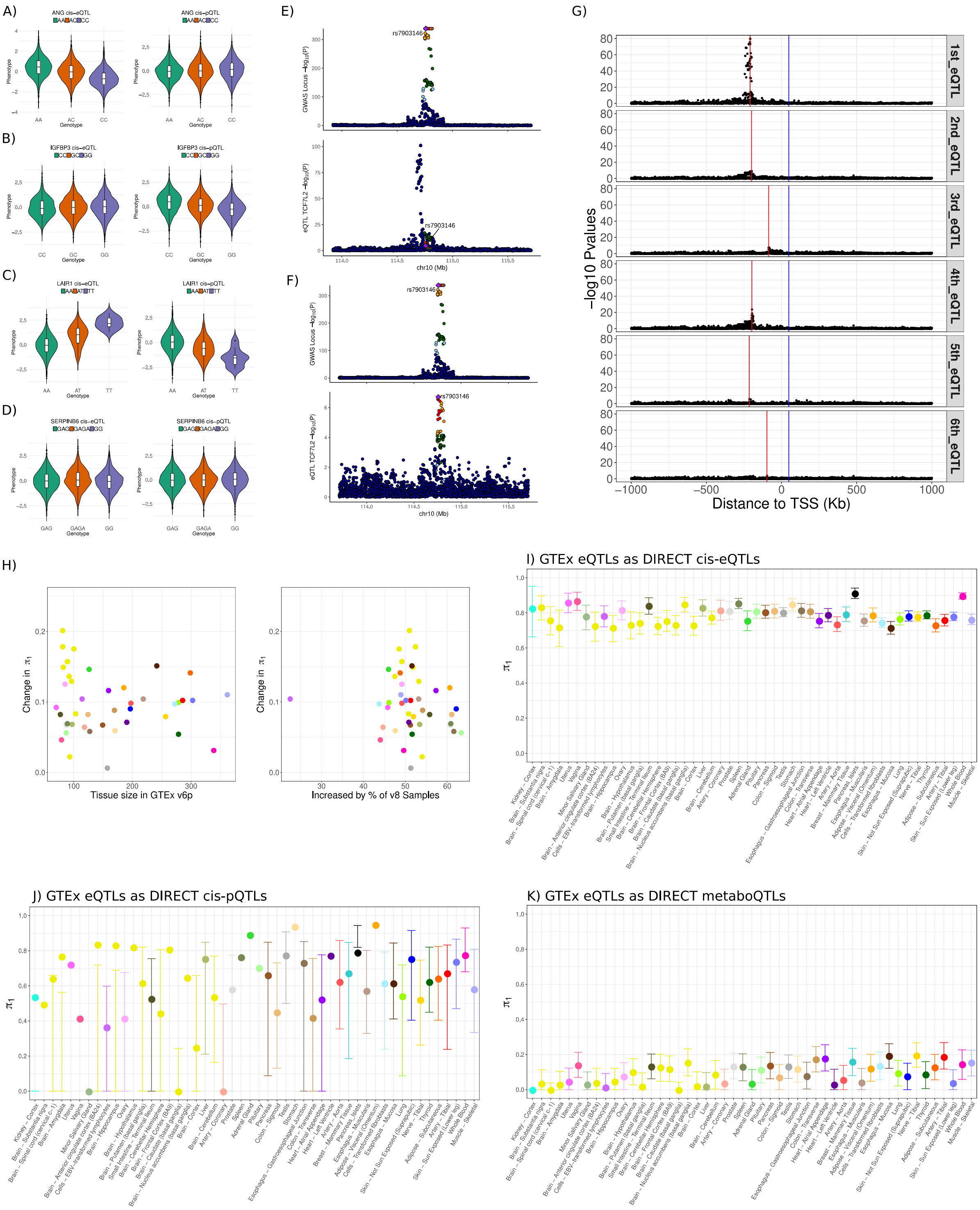

A)
ANG cis-eQTL
ANG cis-pQTL
B)
IGFBP3 cis-eQTL
IGFBP3 cis-pQTL
C)
LAIR1 cis-eQTL
LAIR1 cis-pQTL
D)
SERPINB6 cis-eQTL
SERPINB6 cis-pQTL
Phenotype
Genotype
E)
F)
GWAS Locus -log10(P)
eQTL TCF7L2 -log10(P)
rs7903146
chr10 (Mb)
G)
1st_eQTL
2nd_eQTL
3rd_eQTL
4th_eQTL
5th_eQTL
6th_eQTL
-log10 Pvalues
Distance to TSS (Kb)
H)
Change in π1
Tissue size in GTEx v6p
Increased by % of v8 Samples
I) GTEx eQTLs as DIRECT cis-eQTLs
J) GTEx eQTLs as DIRECT cis-pQTLs
K) GTEx eQTLs as DIRECT metaboQTLs
π1

Supplementary Figure 5 | Shared effects of eQTLs. A – D) Examples of genome-wide significant cis-SNPs regulating gene expression and proteins with opposite direction of effects. The violin plots show the median, first and last quartiles as defined by ggplot[2] geom_violin function. Sample sizes for associations were 3,029 samples for expression and 3,027 for proteins. **E)** Comparative of associations between whole blood expression and T2D using LocusCompare[3]. Top shows associations between SNPs around the TSS of *TCF7L2* with T2D (top, Pvalues of the GWAS association from Mahajan, et al.[4]). Bottom shows Pvalues of the linear associations between the expression of *TCF7L2* in DIRECT whole blood data. The lead GWAS variant (rs7903146) did not associate with the expression of *TCF7L2* in blood. **F)** Comparative of associations between pancreatic islets expression and T2D using LocusCompare[3]. Top shows associations between SNPs around the TSS of *TCF7L2* with T2D (top, Pvalues of the GWAS association from Mahajan, et al.[4]). Bottom shows Pvalues of the linear associations between the pancreatic islets expression (n=420) of *TCF7L2* in pancreatic islets expression form the InsPIRE study (bottom, Viñuela et al,[5]). The lead GWAS variants (rs7903146) was significantly associated with the expression of *TCF7L2* in pancreatic islets. **G)** Regional plot for all the independent cis-eQTLs for *TCF7L2* found in DIRECT blood. A total of 6 Independent cis-eQTLs were significant (FDR<0.05), with the location of their lead eSNPs indicated by red lines. The blue line identify the location of the T2D related GWAS variant (rs7903146). Pvalues were derived from the linear associations between expression and SNPs using n=3,029 samples. **H)** Change in π_1 estimates using GTEx v6p versus v8 eQTLs. The left plot shows the change in π_1 (y-axis) against the tissue sample size in v6p (x-axis), while the left plot shows the change of π_1 against the percentage of sample size increase between GTEx v6p and v8. **I)** Enrichment analysis of GTEx v8 eQTLs discovered as blood eQTLs in DIRECT dataset. Tissues were ordered by sample size from left (smallest) to right (largest). Horizontal bars indicate the 95% confidence interval (C.I.) calculated for π_1 estimates (centre) using bootstrap resampling and the Pvalue distribution of each type of molecular assay tested in another. Number of Pvalues per tissue ranged from 334 in kidney to 14,920 in thyroid. **J)** Enrichment analysis of GTEx v8 eQTLs discovered as blood pQTLs in DIRECT dataset. C.I. were larger for this analyses as the number of overlapping QTLs across tissues oscillates between 13 (kidney) and 311 (Thyroid). Tissues are ordered by sample size from left (smallest) to right (largest). Horizontal bars indicate the 95% confidence interval (C.I.) calculated for π_1 estimates (centre) using bootstrap resampling and the Pvalue distribution of each type of molecular assay tested in another. **K)** Enrichment analysis of GTEx v8 eQTLs discovered as blood metaboQTLs in DIRECT dataset. Tissues are ordered by sample size from left (smallest) to right (largest). Horizontal bars indicate the 95% confidence interval (C.I.) calculated for π_1 estimates (centre) using bootstrap resampling and the Pvalue distribution of each type of molecular assay tested in another. Number of Pvalues per tissues ranged from 4,298 in whole blood to 6,575 in testis.

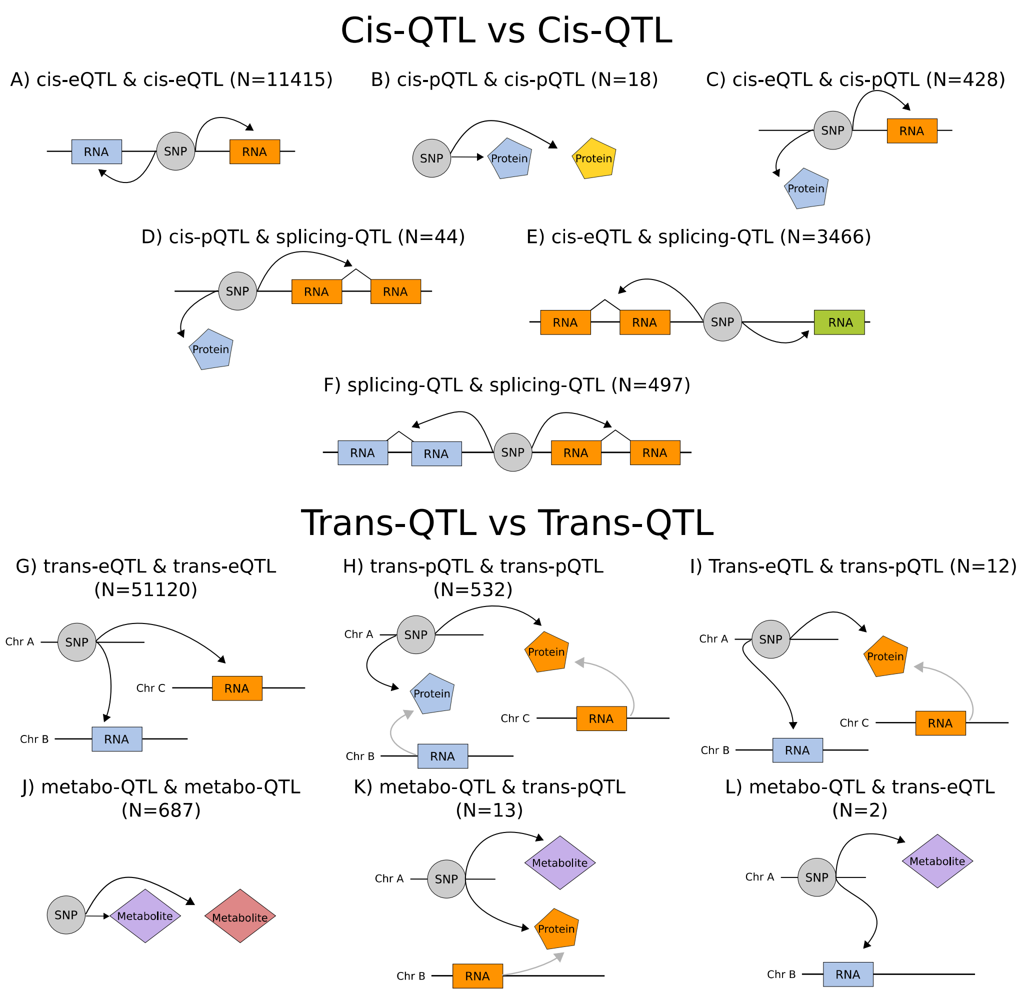

Supplementary Figure 6 | Definition of trios as two molecular phenotypes associated to the same SNP. A – F) Trios of cis-QTLs used to build a network of genetics perturbations across molecular phenotypes. There were 6 possible combinations of cis-QTLs across expression, proteins and splicing phenotypes. **G-L)** Trios of trans-QTLs used to build a network of genetics perturbations across molecular phenotypes. There were 6 possible combinations of trans-QTLs across, considering metabolites-QTLs as trans-QTLs, since they do not have an assigned genomic position.

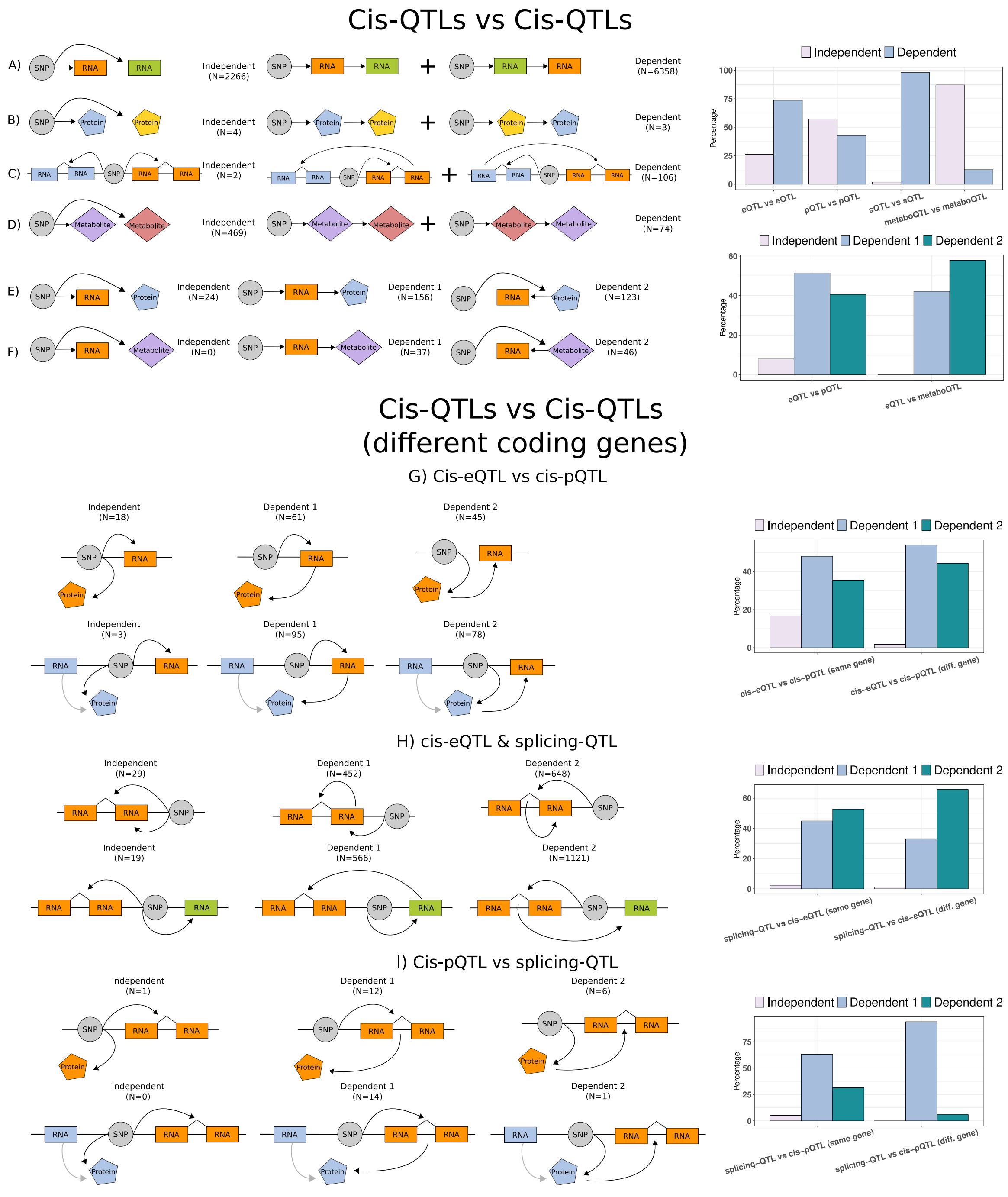

Supplementary Figure 7 | Causal models for shared cis-QTLs. Barplots summarize the numbers indicated in diagrams. These show cartoon representations of the models identified and tested for causality in cis. Colour code for the different models can be interpreted with the cartoons bellow. **A)** cis-eQTLs associated models, were the independent models evaluates unrelated effect of the SNPs in the expression of two genes and dependent models evaluates the dependent effect of the SNP on one gene mediated by the other gene. **B)** Models for cis-pQTLs. **C) M**odels for splicing-QTLs. **(D)** Models for metabolite-QTLs. **E)** Models for shared cis-eQTLs and cis-pQTLs. Here the dependent models distinguish

between the effect of the SNP on proteins mediated by gene expression regulation (Dependent 1), and the effect of the SNP on expression mediated by proteins (Dependent 2). **F)** Models for shared cis-eQTLs and metabo-QTLs. Dependent models show the effect of the SNP on metabolites mediated by gene expression (Dependent 1), and the effect of the SNP on expression mediated by metabolites (Dependent 2). **G)** Models evaluating shared associations between expression and proteins. Top models indicated cases were the SNP affected protein levels and the expression of the coding gene. Here the dependent models distinguish between the effect of the SNP on proteins mediated by gene expression regulation (Dependent 1), and the effect of the SNP on expression mediated by proteins (Dependent 2). **H)** Models evaluating shared associations between expression and splicing. Top models indicated cases were the SNP affected both expression and splicing in the same gene. The dependent models distinguish between the effect of the SNP on gene splicing mediated by gene expression abundance regulation (Dependent 1), and the effect of the SNP on expression abundance mediated by a change in splicing (Dependent 2). **I)** Models evaluating shared associations between proteins and splicing. Top models indicated cases were the SNP affected both protein levels and splicing in the protein coding gene. The dependent models distinguish between the effect of the SNP on gene splicing mediated by protein abundance regulation (Dependent 1), and the effect of the SNP on proteins mediated by a change in splicing (Dependent 2).

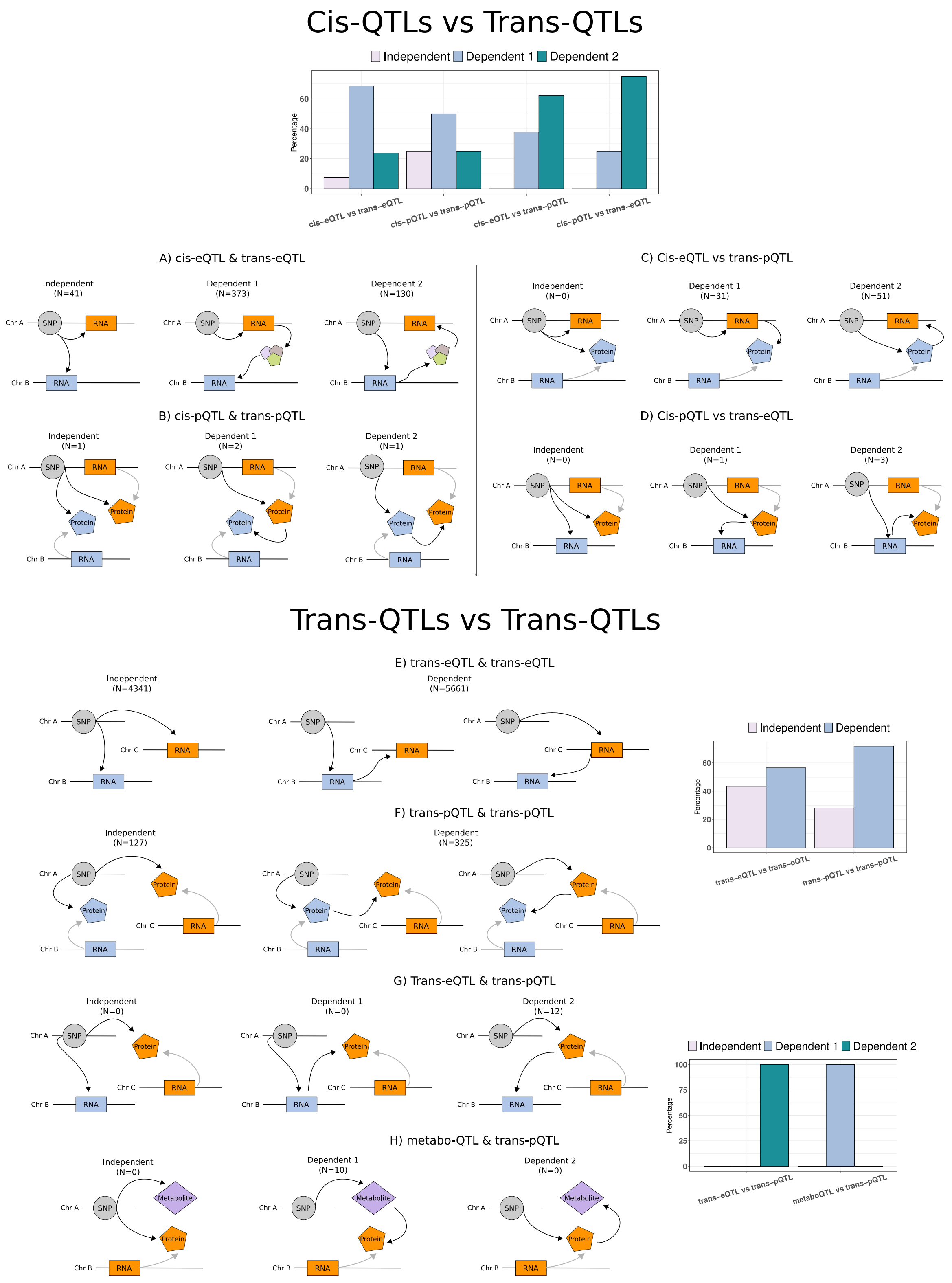

Supplementary Figure 8 | Causal models for shared cis-trans-QTLs and trans-trans-QTLs. The diagram show models used to infer causality for SNPs acting as cis and trans within phenotypes and across different phenotypes. The top barplot summarize the number

of models identified and show on the diagrams. Colour code for the different models can be interpreted with the cartoons bellow. **A)** Number of trios of SNP-phenotypes identified for each model when evaluating SNPs with shared cis and trans-eQTLs effects. Dependent model 1 identifies an effect of the SNP on trans expression mediated by a local effect. This is assumed to be mediated by molecules or proteins not measured. Dependent model 2 identify local effects of the SNP in expression mediated by distal regulation. **B)** Models for SNPs affecting both cis and trans-pQTLs effects. Dependent model 1 identifies an effect of the SNP on distal protein mediated by the effect on a nearby protein. Dependent model 2 identify local effects of the SNP in protein levels mediated by distal regulation of other protein. **C)** Models evaluating SNPs with shared cis-eQTL and trans-pQTLs effects. Dependent model 1 identifies an effect of the SNP on a distal protein in mediated by a local effect on gene expression. Dependent model 2 identify local effects of the SNP in expression mediated by distal regulation of protein levels. **D)** Models evaluating SNPs with shared cis-pQTL and trans-eQTLs effects. Dependent model 1 identifies an effect of the SNP on distal gene expression mediated by a local effect on a protein. Dependent model 2 identify local effects of the SNP in protein abundance mediated by distal regulation of the expression of other gene. **E)** Models for shared trans-eQTL effects. **F)** Models for shared trans-pQTL effects. **G)** Models for shared trans-eQTL and trans-pQTLs effects. The only models confidently identify show the effect of a SNP in distal gene expression were mediated by regulation of distal proteins. **H)** Models evaluating SNPs with shared cis-pQTL and metabo-QTLs effects. The only models identify show the effects of the SNPs on distal proteins were mediated by metabolites.

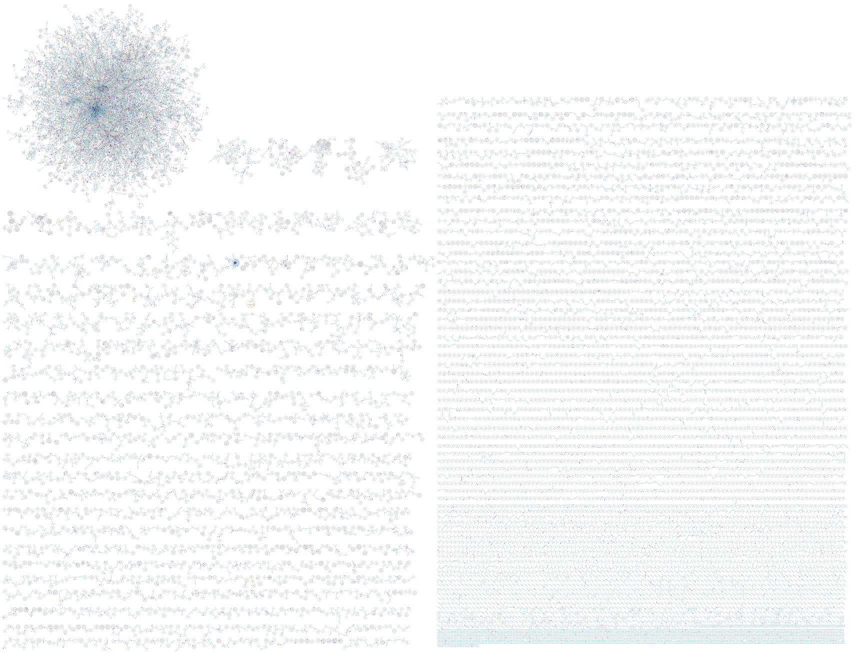

Supplementary Figure 9 | Full network with all QTLs. The figure shows the complete network including all QTLs, including 79,733 nodes (15,254 genes, 373 proteins, 172 metabolites and 63,795 SNPs) and 80,645 edges (QTLs), connected in clusters containing between 3 and 19,711 nodes. Full figure may be explore using a Cytoscape object deposited on the following link: https://zenodo.org/record/7521410

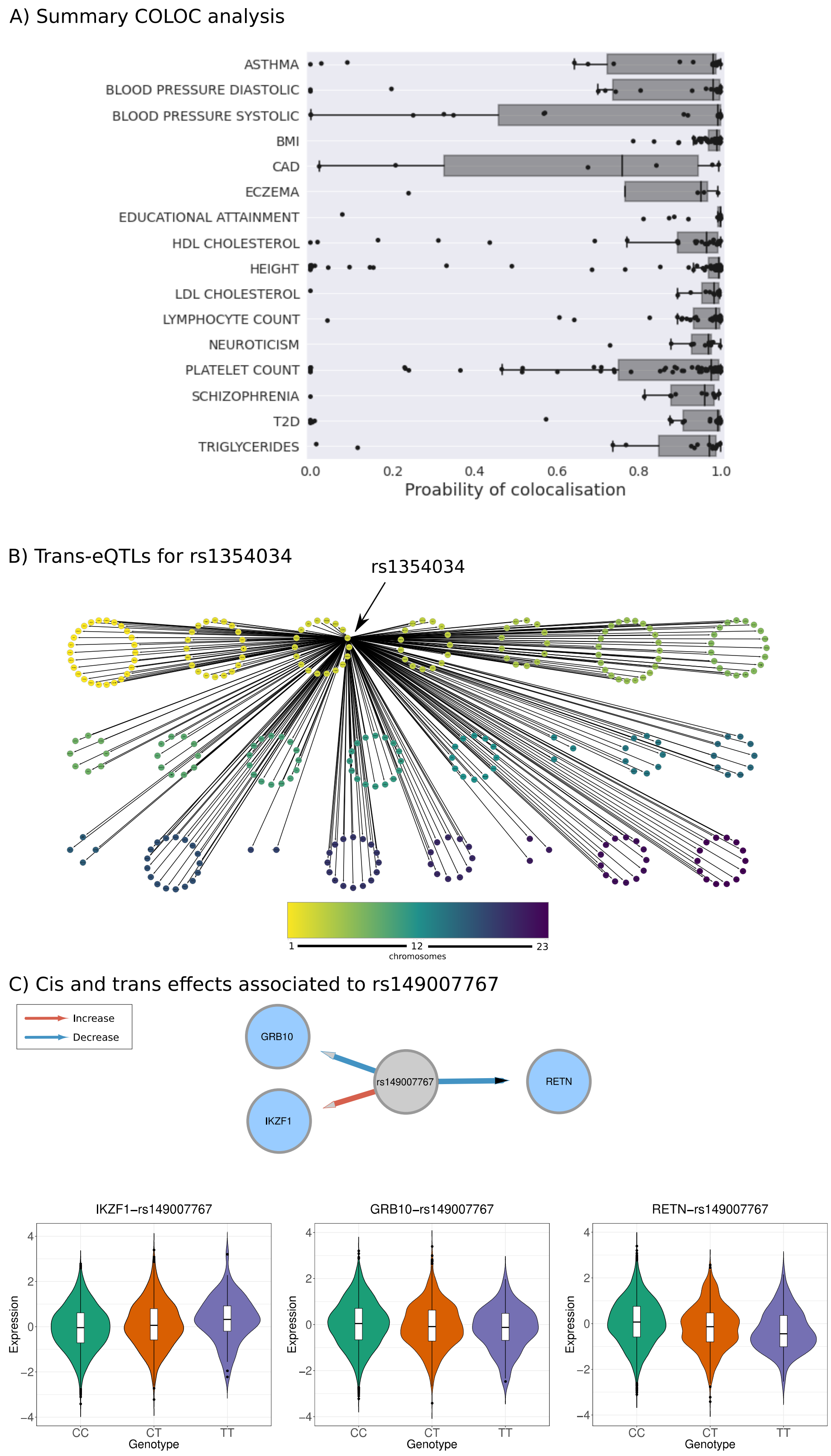
A) Summary COLOC analysis
ASTHMA
BLOOD PRESSURE DIASTOLIC
BLOOD PRESSURE SYSTOLIC
BMI
CAD
ECZEMA
EDUCATIONAL ATTAINMENT
HDL CHOLESTEROL
HEIGHT
LDL CHOLESTEROL
LYMPHOCYTE COUNT
NEUROTICISM
PLATELET COUNT
SCHIZOPHRENIA
T2D
TRIGLYCERIDES
0.0
0.2
0.4
0.6
0.8
1.0
Proability of colocalisation
B) Trans-eQTLs for rs1354034
rs1354034
1
12
23
chromosomes
C) Cis and trans effects associated to rs149007767
Increase
Decrease
GRB10
IKZF1
rs149007767
RETN
IKZF1–rs149007767
GRB10–rs149007767
RETN–rs149007767
Expression
Genotype
CC
CT
TT
4
2
0
−2
−4

Supplementary Figure 10 | Co-localization of different genetic effects. A) Summary of COLOC analysis. Probabilities of co-localization per GWAS SNPs tested (from studies providing complete summary statistics) are shown on the y-axis, with one type of disease/phenotype per row. Boxes show the mean of the probabilities of co-localization across all SNPs tested per trait with the first and last quartiles as defined by ggplot[2]. **B) Trans-eQTLs for rs1354034.** This variant is a trans-eSNP for 297 genes across 23 chromosomes. The diagram shows the 297 genes and the SNP as nodes coloured according to their chromosome location, which are also ordered, with chromosome 1 on the top left, and chromosome X (n=23) on the bottom right. The SNP is located in chromosome 3, and is the origin of all edges. **C) Cis and trans effects associated to rs149007767**. This variant is a trans-eQTL for *RETN* (Pvalue=1.296e-13, beta=-0.134, Figure 5B) while acting as a cis-eQTL for two transcription factors in cis: *GRB10* (Pvalue=1.448e-06; beta=-0.043) and *IKZF1* (Pvalue=1.283e-16; beta=0.566), suggesting the trans-eQTL effect may be mediated by one or two of these genes. Causal inference analysis only found supporting evidence for a model where rs149007767 regulation of *RETN* is mediated by *IKZF1* (BIC > 26, compare with the other two possible models for *RETN*-*IKZF1*-rs149007767. All figures used data from 3,029 independent biological samples, and the violin plots show the median and first and last quartiles as defined by ggplot[2] geom_violin function.

Supplementary References:

1. R Core Team. R: A language and environment for statistical computing. (2018).
2. Wickham, H. *ggplot2*. (Springer International Publishing, 2016). doi:10.1007/978-3-319-24277-4.
3. Liu, B., Gloudemans, M. J., Rao, A. S., Ingelsson, E. & Montgomery, S. B. Abundant associations with gene expression complicate GWAS follow-up. *Nat Genet* **51**, 768–769 (2019).
4. Mahajan, A. *et al.* Fine-mapping type 2 diabetes loci to single-variant resolution using high-density imputation and islet-specific epigenome maps. *Nat Genet* **50**, 1505–1513 (2018).
5. Viñuela, A. *et al.* Genetic variant effects on gene expression in human pancreatic islets and their implications for T2D. *Nature Communications* **11**, 4912 (2020).
