# Supplementary material for: Genetic analysis of blood molecular phenotypes reveals common properties in the regulatory networks affecting complex traits
Source: Nat Commun. 2023 Aug 21;14:5062. doi: 10.1038/s41467-023-40569-3 (PMC10442420; doi:10.1038/s41467-023-40569-3)
Supplement: Supplementary file 3 — Description of Additional Supplementary Files [file 41467_2023_40569_MOESM3_ESM.pdf]

Description of Additional Supplementary Files

File Name: Supplementary Data 1
Description: List of 59,972 significantly independent cis-eQTLs.

File Name: Supplementary Data 2
Description: List of 1,592 significantly independent cis-pQTLs.

File Name: Supplementary Data 3
Description: List of 3,978 significantly independent splicing-QTLs.

File Name: Supplementary Data 4
Description: Functional enrichment results.

File Name: Supplementary Data 5
Description: List of all SNPs significantly associated with two phenotypes in cis and/or trans (n=69,532 trios).

File Name: Supplementary Data 6
Description: List of annotates genes to topologically associated domains (TADs) called in 8 different blood cell types.

File Name: Supplementary Data 7
Description: List of 2,320 significantly independent trans-eQTLs.

File Name: Supplementary Data 8
Description: List of 533 significantly independent trans-pQTLs.

File Name: Supplementary Data 9
Description: List of 301 significantly independent metabolites-QTLs.

File Name: Supplementary Data 10
Description: List of pQTLs and the matching eQTLs.

File Name: Supplementary Data 11
Description: List of replicated QTLs in various studies.

File Name: Supplementary Data 12
Description: Causal model that passed evaluation criteria.

File Name: Supplementary Data 13
Description: List of 3,652 QTLs with a GWAS SNPs.

File Name: Supplementary Data 14
Description: Summary of GWAS SNPs identified as QTLs in this study.

File Name: Supplementary Data 15
Description: List of 16 GWAS studies used in the co-localization analysis.

File Name: Supplementary Data 16
Description: Results from the co-localization analysis (COLOC).

File Name: Supplementary Data 17
Description: STRING enrichment analysis output from the big cluster network from Cytoscape.

File Name: Supplementary Data 18
Description: List of excluded GWAS for enrichment analysis.

File Name: Supplementary Data 19
Description: DIRECT consortium full list.
