# Supplementary material for: Genetic analysis of blood molecular phenotypes reveals common properties in the regulatory networks affecting complex traits
Source: Nat Commun. 2023 Aug 21;14:5062. doi: 10.1038/s41467-023-40569-3 (PMC10442420; doi:10.1038/s41467-023-40569-3)
Supplement: Supplementary file 23 — Reporting Summary [file 41467_2023_40569_MOESM23_ESM.pdf]

Corresponding author(s): Ana Viñuela & Emmanouil T. Dermitzakis

Last updated by author(s): Jun 27, 2023

Reporting Summary

Nature Portfolio wishes to improve the reproducibility of the work that we publish. This form provides structure for consistency and transparency in reporting. For further information on Nature Portfolio policies, see our Editorial Policies and the Editorial Policy Checklist.

Statistics

For all statistical analyses, confirm that the following items are present in the figure legend, table legend, main text, or Methods section.

n/a	Confirmed	
☐	☒	The exact sample size (*n*) for each experimental group/condition, given as a discrete number and unit of measurement
☐	☒	A statement on whether measurements were taken from distinct samples or whether the same sample was measured repeatedly
☐	☒	The statistical test(s) used AND whether they are one- or two-sided<br>*Only common tests should be described solely by name; describe more complex techniques in the Methods section.*
☐	☒	A description of all covariates tested
☐	☒	A description of any assumptions or corrections, such as tests of normality and adjustment for multiple comparisons
☐	☒	A full description of the statistical parameters including central tendency (e.g. means) or other basic estimates (e.g. regression coefficient) AND variation (e.g. standard deviation) or associated estimates of uncertainty (e.g. confidence intervals)
☐	☒	For null hypothesis testing, the test statistic (e.g. *F*, *t*, *r*) with confidence intervals, effect sizes, degrees of freedom and *P* value noted<br>*Give P values as exact values whenever suitable.*
☐	☒	For Bayesian analysis, information on the choice of priors and Markov chain Monte Carlo settings
☒	☐	For hierarchical and complex designs, identification of the appropriate level for tests and full reporting of outcomes
☒	☐	Estimates of effect sizes (e.g. Cohen's *d*, Pearson's *r*), indicating how they were calculated

Our web collection on statistics for biologists contains articles on many of the points above.

Software and code

Policy information about availability of computer code

Data collection	No software was used for data collection.
Data analysis	Software used for analyses: SHAPEIT v2.r790; IMPUTE v2.3.2; FastQTL v1; COLOC v2.

For manuscripts utilizing custom algorithms or software that are central to the research but not yet described in published literature, software must be made available to editors and reviewers. We strongly encourage code deposition in a community repository (e.g. GitHub). See the Nature Portfolio guidelines for submitting code & software for further information.

Data

Policy information about availability of data

All manuscripts must include a data availability statement. This statement should provide the following information, where applicable:

- Accession codes, unique identifiers, or web links for publicly available datasets
- A description of any restrictions on data availability
- For clinical datasets or third party data, please ensure that the statement adheres to our policy

The molecular and clinical raw data as well as the processed are available under restricted access due to the informed consent given by study participants, the various national ethical approvals for the present study, and the European General Data Protection Regulation (GDPR), individual-level clinical and molecular data cannot be transferred from the centralized IMI-DIRECT repository. Requests for access will be informed on how data can be accessed via the DIRECT secure analysis platform following submission of an appropriate application. The IMI-DIRECT data access policy is available at https://directdiabetes.org. As described in the

methods section we used the human genome build GRCh37 as a reference for genomic location of genotypes and transcriptomics data, and Gencode v19 54 for gene models and TSS information (https://www.gencodegenes.org/human/release_19.html). For functional enrichment analyses we used dataset from the Ensembl Variant Effect Predictor14 (VEP) information v98 (https://grch37.ensembl.org/info/docs/tools/vep/script/vep_download.html) and ChromHMM13 models (https://egg2.wustl.edu/roadmap/data/byFileType/chromhmmSegmentations/ChmmModels/imputed12marks/jointModel/final/). GTEx v6p and v8 summary statistics were accessed using the GTEx Portal (https://www.gtexportal.org/home/). The 16 GWAS studies evaluated for co-localization and the links to their summary statistics are listed in Supplementary Data 15.
Complete summary statistics including cis and trans genetic associations for gene expression, proteins and metabolites, as well as files to visualize networks on Cytoscape are freely available in the following link https://zenodo.org/record/7521410

Research involving human participants, their data, or biological material

Policy information about studies with human participants or human data. See also policy information about sex, gender (identity/presentation), and sexual orientation and race, ethnicity and racism.

Reporting on sex and gender	Sex was considered in the study design as a covariate and included in all analyses to control for sex differences between individuals. We did not derive any specific finding associated to either sex or gender. Sex was determined using genetic information in agreement with expression of the XIST gene in RNAseq data and self-reports. Any inconsistency between the 3 sources of information resulted in the removal of all data associated to the sample. Gender was not considered for analyses.
Reporting on race, ethnicity, or other socially relevant groupings	We did not collect, employ or derive any results from social categorization variables. In genetic analyses we included 3 principal components derived from genotype data to control for potential population structure.
Population characteristics	The DIRECT (Diabetes Research on Patient Stratification) consortium includes pre-diabetic participants (target sample size 2,200–2,700) and patients with newly diagnosed type 2 diabetes (target sample size ~1,000) with detailed metabolic phenotyping and European descent. The cohort included 2,142 men and 887 women with a mean age of 61.6 years old (yo), and a range of 30yo to 75yo. Data from both group of participants were used during the analyses as one group and controlling for their diabetic status, sex and age when appropriate.
Recruitment	Participants for the IMI DIRECT cohort of individuals with a pre-diabetic status were recruited from an existing large sample frame (N = 24,682) derived from established prospective cohort studies across European institutions. Individuals with a newly diagnosed diabetes status used clinical registries to identify eligible participants. Given the type of analyses used in this study, we do not believe the recruitment of participants can influenced our results.
Ethics oversight	Ethics approval for the study protocol was obtained by all the regional research ethics review boards (Lund, Sweden: 20130312105459927; Copenhagen, Denmark: H-1-2012-166 and H-1-2012-100; Amsterdam, Netherlands: NL40099.029.12; Newcastle, Dundee, and Exeter, UK: 12/NE/0132).

Note that full information on the approval of the study protocol must also be provided in the manuscript.

Field-specific reporting

Please select the one below that is the best fit for your research. If you are not sure, read the appropriate sections before making your selection.

☒ Life sciences ☐ Behavioural & social sciences ☐ Ecological, evolutionary & environmental sciences

For a reference copy of the document with all sections, see nature.com/documents/nr-reporting-summary-flat.pdf

Life sciences study design

All studies must disclose on these points even when the disclosure is negative.

Sample size	3,029 samples were available across studies after quality assessment.
Data exclusions	After quality assessment, no samples were removed form the study. For simplicity, the causal network analyses were done in 3,027 samples, removing all data associated to the samples from two individuals from which protein data not available.
Replication	We performed replications of the eQTLs, pQTLs and metabolites-QTLs. For eQTLs in cis and trans we used the GTEx datasets across 53 tissues in total and eQTLGen. We were able to evaluate 514 gene-SNP pairs from DIRECT trans-eQTLs, of which 463 were also significant. For cis and trans-pQTLs replication we used GWAS summary statistics from Sun et al. (PMID: 29875488) and found that 281 cis-pQTL and 65 trans-pQTLs affecting 253 proteins replicated. For metabolites, we were able to evaluate 65 metabolite-SNPs pairs from 47 metabolites, of which all of them replicated in Long et al. (PMID: 28263315). Causal network analyses is not possible to replicate and no other data-set offer the information required for these analyses.
Randomization	Data were generated in experiments that randomize the position of the samples within each of the two cohort on the plates used for proteins and targeted metabolites measurements. Untargeted metabolites, genotypes and transcriptomics data randomize samples across all 3,029 samples.
Blinding	Blinding is not relevant to this study since all data types were derived from anonymised samples and using randomizations during data generations.

Behavioural & social sciences study design

All studies must disclose on these points even when the disclosure is negative.

Study description

Research sample

Sampling strategy

Data collection

Timing

Data exclusions

Non-participation

Randomization

Ecological, evolutionary & environmental sciences study design

All studies must disclose on these points even when the disclosure is negative.

Study description

Research sample

Sampling strategy

Data collection

Timing and spatial scale

Data exclusions

Reproducibility

Randomization

Blinding

Did the study involve field work? ☐ Yes ☐ No

Field work, collection and transport

Field conditions

Location

Access & import/export

Disturbance

Reporting for specific materials, systems and methods

We require information from authors about some types of materials, experimental systems and methods used in many studies. Here, indicate whether each material, system or method listed is relevant to your study. If you are not sure if a list item applies to your research, read the appropriate section before selecting a response.

Materials & experimental systems

n/a	Involved in the study
☒	☐ Antibodies
☒	☐ Eukaryotic cell lines
☒	☐ Palaeontology and archaeology
☒	☐ Animals and other organisms
☒	☐ Clinical data
☒	☐ Dual use research of concern
☒	☐ Plants

Methods

n/a	Involved in the study
☒	☐ ChIP-seq
☒	☐ Flow cytometry
☒	☐ MRI-based neuroimaging

Antibodies

Antibodies used

Validation

Eukaryotic cell lines

Policy information about cell lines and Sex and Gender in Research

Cell line source(s)

Authentication

Mycoplasma contamination

Commonly misidentified lines
(See ICLAC register)

Palaeontology and Archaeology

Specimen provenance

Specimen deposition

Dating methods

☐ Tick this box to confirm that the raw and calibrated dates are available in the paper or in Supplementary Information.

Ethics oversight

Note that full information on the approval of the study protocol must also be provided in the manuscript.

Animals and other research organisms

Policy information about studies involving animals; ARRIVE guidelines recommended for reporting animal research, and Sex and Gender in Research

Laboratory animals

Wild animals

Reporting on sex

Field-collected samples

Ethics oversight

Note that full information on the approval of the study protocol must also be provided in the manuscript.

Clinical data

Policy information about clinical studies

All manuscripts should comply with the ICMJE guidelines for publication of clinical research and a completed CONSORT checklist must be included with all submissions.

Clinical trial registration

Study protocol

Data collection

Outcomes

Dual use research of concern

Policy information about dual use research of concern

Hazards

Could the accidental, deliberate or reckless misuse of agents or technologies generated in the work, or the application of information presented in the manuscript, pose a threat to:

No	Yes	
☐	☐	Public health
☐	☐	National security
☐	☐	Crops and/or livestock
☐	☐	Ecosystems
☐	☐	Any other significant area

Experiments of concern

Does the work involve any of these experiments of concern:

No	Yes	
☐	☐	Demonstrate how to render a vaccine ineffective
☐	☐	Confer resistance to therapeutically useful antibiotics or antiviral agents
☐	☐	Enhance the virulence of a pathogen or render a nonpathogen virulent
☐	☐	Increase transmissibility of a pathogen
☐	☐	Alter the host range of a pathogen
☐	☐	Enable evasion of diagnostic/detection modalities
☐	☐	Enable the weaponization of a biological agent or toxin
☐	☐	Any other potentially harmful combination of experiments and agents

Plants

Seed stocks

Novel plant genotypes

Authentication

ChIP-seq

Data deposition

☐ Confirm that both raw and final processed data have been deposited in a public database such as GEO.

☐ Confirm that you have deposited or provided access to graph files (e.g. BED files) for the called peaks.

Data access links
May remain private before publication.

Files in database submission

Genome browser session
(e.g. UCSC)

Methodology

Replicates

Sequencing depth

Antibodies

Peak calling parameters

Data quality

Software

Flow Cytometry

Plots

Confirm that:

☐ The axis labels state the marker and fluorochrome used (e.g. CD4-FITC).

☐ The axis scales are clearly visible. Include numbers along axes only for bottom left plot of group (a 'group' is an analysis of identical markers).

☐ All plots are contour plots with outliers or pseudocolor plots.

☐ A numerical value for number of cells or percentage (with statistics) is provided.

Methodology

Sample preparation

Instrument

Software

Cell population abundance

Gating strategy

☐ Tick this box to confirm that a figure exemplifying the gating strategy is provided in the Supplementary Information.

Magnetic resonance imaging

Experimental design

Design type

Design specifications

Behavioral performance measures

Acquisition

Imaging type(s)

Field strength

Sequence & imaging parameters

Area of acquisition

Diffusion MRI ☐ Used ☐ Not used

Preprocessing

Preprocessing software

Normalization

Normalization template

Noise and artifact removal

Volume censoring

Statistical modeling & inference

Model type and settings

Effect(s) tested

Specify type of analysis: ☐ Whole brain ☐ ROI-based ☐ Both

Statistic type for inference

(See Eklund et al. 2016)

Correction

Models & analysis

n/a	Involved in the study
☐	☐ Functional and/or effective connectivity
☐	☐ Graph analysis
☐	☐ Multivariate modeling or predictive analysis

Functional and/or effective connectivity

Graph analysis

Multivariate modeling and predictive analysis
